# Healing effect of *Dillenia indica* fruit extracts standardized to betulinic acid on ultraviolet radiation-induced psoriasis-like wounds in rats

**DOI:** 10.1080/13880209.2016.1266672

**Published:** 2016-12-12

**Authors:** Maicon Roberto Kviecinski, Isabela Machado Barbosa David, Flávia de Souza Fernandes, Marina dos Reis Correa, Morgana Miranda Clarinda, Amanda Fernandes Freitas, Jane da Silva, Marta Gava, Simony Davet Müller, Drielly Florentino, Fabrícia Petronilho, Diego Moterle, Luiz Alberto Kanis, Rozangela Curi Pedrosa

**Affiliations:** a Post-Graduate Program in Health Sciences, Universidade do Sul de Santa Catarina, Palhoça, Brazil;; b Pharmacy School, Universidade do Sul de Santa Catarina, Tubarão, Brazil;; c Veterinary School, Universidade do Sul de Santa Catarina, Tubarão, Brazil;; d Department of Biochemistry, Universidade Federal de Santa Catarina, Florianópolis, Brazil

**Keywords:** Plant, skin, topical treatment, anti-inflammatory, antioxidant

## Abstract

**Context:** *Dillenia indica* Linn. (Dilleniaceae) is traditionally used to treat skin inflammation.

**Objective:** This study evaluated the healing effect of *Dillenia indica* fruit extracts on induced psoriasis-like wounds in Wistar rats.

**Materials and methods:** Extracts were standardized to betulinic acid, including an aqueous ethanolic extract (AEE), ethyl acetate extract (EAE) and petroleum ether extract. Effects against lipid peroxidation were assessed *in vitro*. Wounds were created at rat tails (*n* = 12). Topical treatments were applied once daily for 7 days (1 mL of AEE or EAE at 5 or 50 mg/mL). Maximal dose was defined by the extract solubility. A 10-fold lower dose was also tested. Positive and negative controls were treated with clobetasol (0.5 mg/mL) or excipient. Half of each group was euthanized for histology. The remaining animals were observed for 20 days for wound measurements.

**Results:** Yields of AEE and EAE were 4.3 and 0.7%, respectively. Betulinic acid concentrations in AEE and EAE were 4.6 and 107.6 mg/g. Extracts neutralized lipid peroxidation *in vitro* at 0.02 μg/mL, accelerating healing at 50 mg/mL. Complete healing in mice treated with AEE occurred 16 days after wound induction. This time was 14 and 12 days in mice treated with EAE and clobetasol. Compared to orthokeratosis, parakeratosis was reduced by AEE (25%), EAE (45%) and clobetasol (55%). EAE caused superior protection against biomolecules oxidation of skin compared to AEE.

**Discussion and conclusion:** EAE exhibited activity closer to that of clobetasol. Betulinic acid may be an active constituent, which should be assessed in future studies.

## Introduction

*Dillenia indica* Linn. (Dilleniaceae) is an evergreen tree originally from tropical Asia that is well distributed in other countries, such as India, Indonesia, Sri Lanka, Malaysia, Thailand and Vietnam. This plant is known primarily as ‘elephant-apple’ because the fruits are relished by elephants, which are important seed dispensers for this tree (Apu et al. 2010; Gandhi & Mehta 2013). *Dillenia indica* (*D. indica*) is known as ‘árvore-da-pataca’ or ‘dilênia’ in Brazil, and it was introduced during the nineteenth century (USDA/ARS 1995). The plant grows well in the country’s coastal regions.

Cultural aspects distinguish the natural relationships between plants and people in Asia, where *D. indica* is native, and Brazil, where it was introduced. This tree possesses a large crown and beautiful white aromatic flowers, and it has been used in Brazil primarily for ornamental purposes (Lorenzi et al. 2003). The entire plant is used for medicinal purposes in Asia, and the fruits are used in the cuisine (Gandhi & Mehta 2013).

Asians use the aerial parts of *D. indica* in oral or topical preparations to treat abdominal and joint pain, cough, diarrhoea, fever, tumours, diabetes, toning up the nervous system and removing fatigue (Shome et al. 1980; Sharma et al. 2001; Sood et al. 2005; Yeshwante et al. 2009). Most traditional uses of *D. indica* in folk medicine are associated with anti-inflammatory purposes (Yeshwante et al. 2009). Popular allegations in Brazil corroborate this use, particularly the fruits, which are used in preparations for skin applications to treat inflammation (Lorenzi et al. 2003; Franco 2012).

*Dillenia indica* was investigated scientifically, and these studies demonstrated numerous pharmacological activities, such as anti-inflammatory, antimicrobial, antidiabetic, hypolipidemic and antidiarrhoeal effects of extracts from leaves (Yeshwante et al. 2009; Apu et al. 2010; Kumar et al. 2011; Rahman et al. 2011; Khare et al. 2013). Antinoceptive and antioxidant activities were demonstrated for the methanolic extract of *D. indica* bark (Alam et al. 2012), and anti-leucemic, anti-diarrhoeal and anti-inflammatory actions were demonstrated for extracts from the fruit of *D. indica* (Kumar et al. 2010; Migliato et al. 2011; Rahman et al. 2011).

The literature reveals that different parts of *D. indica* contain many primary and secondary metabolites. The plant is a rich source of triterpenoids, flavonoids, tannins and other less abundant constituents (Gandhi & Mehta 2013). The rich content of flavonoids and triterpenoids in a fraction from leaves exhibited anti-inflammatory activity (Khare et al. 2013). However, most of these previous studies did not evaluate standardized extracts or isolated substances, which makes it difficult to infer the role of any particular plant constituent.

Kumar et al. (2010) standardized the methanol extract from the fruits of *D. indica* to betulinic acid, which is a natural lupane-type pentacyclic triterpenoid. This acid was considered one of the major bioactive compounds in a study that reported antileukemic activity. Betulinic acid was also considered responsible for anti-inflammatory effects (Jingbo et al. 2015; Lingaraju et al. 2015). Therefore, this study assessed the healing effect of different extracts from the fruit of *D. indica* standardized to betulinic acid on ultraviolet (UV) radiation-induced psoriasis-like wounds in rats. The Perry’s scientific mouse tail model is accepted for investigations of anti-psoriatic activity (Vijayalakshmi et al. 2012). Potential protection against oxidative damage in constitutive biomolecules of the skin was evaluated.

## Materials and methods

### Plant material

Fruits of *D. indica* were harvested in the gardens of the Universidade do Sul de Santa Catarina (Unisul) in Tubarão, Santa Catarina, Brazil (July 2013). Professor Jasper José Zanco from the same University evaluated plant authenticity. A voucher specimen was deposited at Herbarium *Laelia purpurata* in Unisul (SRS5103). This study respected all rules of biological biodiversity.

### Extraction

Fresh ripe fruits were milled and maintained in shake-assisted macerators in a solvent at 1:2 (w/v) for 2 days, which was repeated three times. Three solvents were used to produce an aqueous ethanolic extract (AEE) made with water:ethanol (1:8), an ethyl acetate extract (EAE) made with ethyl acetate alone (≥99%) and a petroleum ether extract (PEE). All solvents were Vetec^®^ ACS grade reagents. Values of dielectric constants (*ɛ*) assumed for ethyl acetate (6.02), petroleum ether (4.03), water (78.36) and ethanol (24.30) were based on the literature. The dielectric constant of the mixture water:ethanol (1:8) was calculated using the ratio *ɛ* = *ɛ*_w_ . *f*_w_ + *ɛ*_e_ . *f*_e_, with *ɛ*_w_ and *ɛ*_e_ representing the dielectric constants of water and ethanol, respectively, and *f*_w_ and *f*_e_ their volume fraction, respectively (Lund 2009). Solvents were eliminated under reduced pressure after extractions. Extraction performance was calculated in terms of dried extract yield (%), based on the mass of the starting material (Handa 2008).

### Quantification of betulinic acid

Betulinic acid in the extracts was quantified using high-performance liquid chromatography (HPLC) in a Shimadzu chromatography workstation (Shimadzu, Japan) assembled with a pump Shimadzu LC10AD, CLASS-agent data management software, a Shimadzu SPD-10A HPLC-detector (UV/VIS) and a Shimadzu CTO10AS VP oven. The protocol was based on previously described methods (Oliveira et al. 2002; Kumar et al. 2010). HPLC grade solvents were obtained from Vetec Química Fina Ltda. Precisely weighed samples of extracts were dissolved in methanol (AEE at 5 mg/mL and EAE at 10 mg/mL) in an ultrasonic bath (Thornton^®^, Brazil) and passed through a 0.45 μm filter (Millipore, Billerica, MA). Aliquots of 20 μL were injected onto a C_18_ column (4.6 × 150 mm, 5 μm particle size, Phenomenex Luna^®^, Torrance, CA). The mobile phase was acetonitrile:phosphoric acid 0.25% (9:1), pH 3.0, which was pumped at a constant flow rate of 1 mL/min (isocratic elution). Eluates were monitored at 210 nm. This analytical method was validated previously. A calibration curve (not shown) was constructed using standard solutions of betulinic acid (Sigma-Aldrich Cat. 91466, ≥97.0). Linear regression analysis data for the calibration plots revealed a good linear relationship, with an *r* ^2 ^=^ ^0.9994 in the concentration range of 25–300 μg/mL with respect to the peak area. The repeatability (1.2%) of this analytical method was performed using six replicates of the same sample, and the results are expressed in percentages relative to the standard deviation. The detection and quantification limits were 0.075 and 0.25 μg/mL, respectively. Recovery percentages from AEE and EAE were 97.3 ± 3.4% and 98.8 ± 2.9%, respectively. Therefore, the method was considered acceptable under the stated operational conditions. Data are expressed in mg of betulinic acid per gram of extract.

### Phytochemical screening of extracts

Precipitation reactions were used for the identification of alkaloids. Extracts (2 mg) were dissolved in water (1 mL), and 2–3 drops of Mayer’s reagent (1.4% HgCl_2_ and 5% KI) or Bouchardat’s reagent (2% iodine and 4% KI) were added (Shrivastav et al. 2009). Flavonoids were detected using the Pew’s reaction (Peach & Tracey 1956). Steroids and triterpenes were assessed using the Liebermann–Burchard test (Shrivastav et al. 2009). Anthraquinones and anthracenes were evaluated using the Borntraeger reaction (Christensen & Abdel-Latif 1949).

### Lipid peroxidation test *in vitro*

Egg yolk was used as a source of lipids (Petronilho et al. 2012; Müller et al. 2016). Yolks were homogenized (1% w/v) in 20 mM phosphate buffer (pH 7.4). The free radical-generating compound 2,2′-azobis(2-methylpropionamidine) dihydrochloride (AAPH, Aldrich Cat. 440914) was used at 120 μM (1:9 v/v) to induce lipid peroxidation. Extracts were added to the reaction mixture at 0.02 to 2 μg/mL. Clobetasol propionate (Sigma-Aldrich Cat. C8037) was used at 100 μM (46.7 μg/mL) as the positive control (Jaques et al. 2012). Reactions were performed for 30 min at 37 °C. Aliquots of each sample (0.5 mL) were centrifuged with 0.5 mL of 15% trichloroacetic acid (Sigma-Aldrich Cat. T6399, Torrance, CA) at 1200 *g* for 10 min. The supernatants (0.5 mL) were mixed with 0.5 mL of 0.67% thiobarbituric acid (Sigma-Aldrich Cat. T5500) and heated at 95 °C for 30 min (Scola et al. 2011). This reaction generated thiobarbituric acid reactive substances (TBARS), which are formed as by-products of lipid peroxidation. Malondialdehyde (MDA) is one of several end products formed via the decomposition of certain lipid peroxidation products. The absorbance was measured at 532 nm (Scola et al. 2011). Data are expressed as concentrations (nmol) of MDA normalized by the protein content (Lowry et al. 1951).

### Animals

The Animal Care and Use Committee from the Universidade do Sul de Santa Catarina approved this protocol (13.032.4.03.IV). All animal experiments were performed in accordance with the National Institutes of Health (NIH) guide for the care and use of laboratory animals (NIH Publication No. 80-23; revised 1978). Male albino Wistar rats (250 ± 20 g) were housed under controlled conditions (12 h light/dark cycle, 22 ± 2 °C, 60% air humidity) and had free access to food and water. Animals were acclimatized for at least 5 days before experiments.

### Wound induction and treatment

The upper medial part of the rat tail (5 cm) was irradiated for 30 min (1.5 J/cm^2^) at a vertical distance of 20 cm with UV lamps (Philips, Brazil) to induce skin wounds (day zero) (Vogel & Vogel 2002). Rats were divided into six groups (*n* = 12): a negative control treated with excipient (1 mL of ethanol:water 1:2) and a positive control treated with clobetasol propionate (Sigma-Aldrich Cat. C8037) at 0.5 mg/mL (1 mL). Clobetasol is a corticosteroid anti-inflammatory drug that is used to treat psoriasis (Gordon 1998). Four test groups were treated with AEE or EAE at 5 mg/mL or 50 mg/mL (1 mL), respectively. The maximal dose was defined by the solubility of the extracts in the excipient. Extracts were difficult to dissolve in formulations containing >50 mg/mL. A 10-fold lower dose of each extract was also tested. The excipient was chosen based on the macerations of *D. indica* fruits that are generally used traditionally and compatibility with clobetasol (Bruze et al. 1995). Treatments were applied topically to the wound area. No signs of toxicity, such as death, hair erection, dullness, inactivity or loss of appetite, were observed. Treatments were initiated 72 h after wound induction, when wounds were apparent, and repeated every 24 h for 7 days.

### Wound-healing assessment

Wound healing was monitored using macroscopic and microscopic evaluations. Half of each group (*n* = 6) was euthanized 24 h after the last dose, and tails were amputated for histology and measurements of biomarkers of oxidative damage in the biomolecules of the skin. The remaining animals were kept alive (*n* = 6/each group) for daily observation for 20 days to quantify the time required for healing. Pictures were taken using a digital camera (Kodak Easy Share CX7430, Rochester, NY). Images were analyzed using CHPTool 5.0 software (Cyclops Group, Brazil), as previously described (Martins et al. 2011). Wound healing was determined using the following formula:

Woundhealing(%) = (Healed area×100)/Wounded area

### Histology

**Figure 1.**
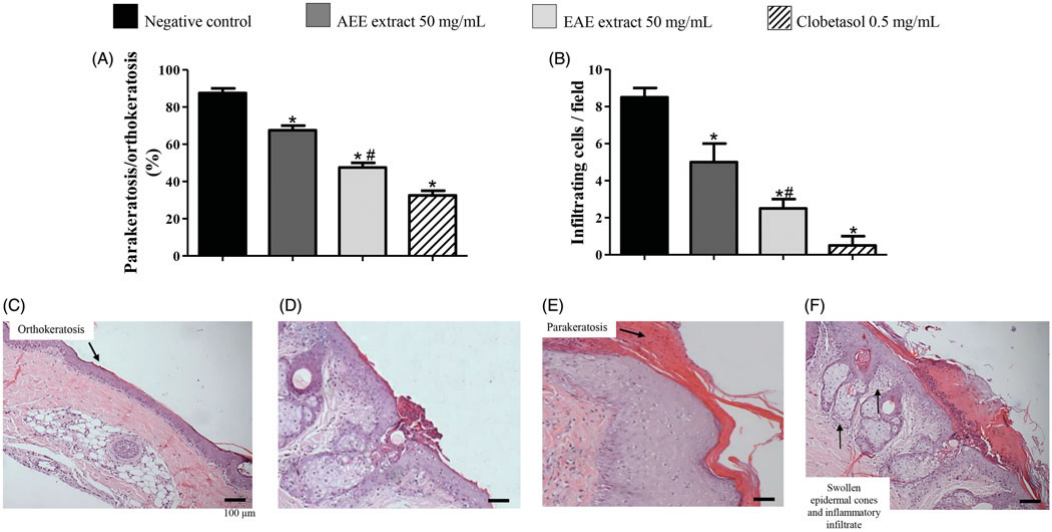
Data of microscopic evaluations (histology) of skin from the tails of rats with radiation-induced wounds that were treated with 50 mg/mL of AEE or EAE from the fruit of *D. indica* L. and the control groups. The analysis was performed in technical triplicates with samples of tissue of six mice from each group (biological replicates). The positive control was treated with clobetasol propionate (0.5 mg/mL), and the negative control was treated only with the excipient (1 mL of ethanol:water 1:2). (A) Percentage of parakeratosis compared to orthokeratosis and (B) quantification of infiltrating inflammatory cells. (C) Image of a slice of tissue from a positive control showing no parakeratosis (score 1) and, predominant orthokeratosis instead. (D) Images of tissues used to grade parakeratosis in comparison to orthokeratosis: minimum (score 2), (E) moderate (score 3). (F) Image of a slice of tissue from a negative control with intense parakeratosis (score 4) and swollen epidermal cones with inflammatory infiltrate. 400 × magnification (*) denotes significant difference compared to the negative control, *p* < 0.001. (#) denotes significant difference compared to AEE treatment, *p* < 0.05.

Cutaneous tissues were fixed in formaldehyde 10% (24 h). Tissue was embedded in paraffin, sliced vertically using a microtome (Leica Biosystems RM2235, Germany) and stained with haematoxylin (Merck Cat. 115938) and eosin (Merck Cat 115935) (Yuan et al. 2010). Slides were examined under a Olympus CX41 microscope, and a Olympus Image Analysis Software (Olympus, Japan) was used to quantify the following factors: (a) Parakeratosis, which is a keratinization that is characterized by an aberrant retention of nuclei in the stratum corneum; (b) Orthokeratosis, which is the formation of a nuclear keratin layer in the normal epidermis and (c) quantification of infiltrating inflammatory cells. The analysis was performed in technical triplicates with tissue samples of six mice from each group (biological replicates) (Vijayalakshmi et al. 2012). Parakeratosis and orthokeratosis were quantified using an adaptation of the method described previously by Wolberink et al. (2011). The surface percentage of total horizontal involvement of parakeratosis compared to orthokeratosis was first estimated visually and categorized in four groups: zero, <30, between 30 and 70 and >70 up to 100% (Figure 1(C–F), respectively). The analysis was carried out always by the same observer. Then, a score value was given respectively to each category in the 1–4 range (1 – absent, 2 – minimum, 3 – moderate and 4 – intense), resulting in a mean score for each group. The results were expressed in percentages compared to the negative control, being the value of score 4 the representation of maximal predominance of parakeratosis over orthokeratosis.

### Biomarkers of oxidative damage in biomolecules of tail skin

Lipid peroxidation was measured using the TBARS reaction (Ohkawa et al. 1979). Homogenates of fresh skin tissue from the rat tails were prepared in buffer (30 μM Na_2_PO_4_ and 140 μM KCl, pH 7.4) at 1:2 (w/v). Samples were centrifuged (10 min, 1000 *g*), and supernatants were mixed with 10% trichloroacetic acid (1:3) (Sigma-Aldrich Cat. T6399), and centrifuged. Thiobarbituric acid (0.67%, 1:2 v/v) (Sigma-Aldrich Cat. T5500) was added, and the solution was heated (95 °C, 30 min). Absorbances were read at 532 nm. Carbonyl proteins were measured in homogenates prepared in buffer (40 μM KH_2_PO_4_ and 120 μM KCl pH 7.4). Carbonyl groups in the sample react covalently with 2,4-dinitrophenylhydrazine (Aldrich, Cat. D199303) to form a 2,4-dinitrophenylhydrazone, which is quantifiable at 370 nm (Levine et al. 1990). These assays were performed in technical triplicates with tissue samples of six mice from each group (biological replicates). All data were normalized by protein content (Lowry et al. 1951).

### Statistical analysis

Data are expressed as the means ± standard deviation or percentages. The assays were performed in technical triplicates. Data were analyzed using analysis of variance (ANOVA) and the Bonferroni test. Comparisons and differences were processed using GraphPad Prism software (San Diego, CA). Values of *p* < 0.05 were considered statistically significant.

## Results and discussion

Table 1 shows some features of the initial extraction procedures, such as the solvents used to prepare the extracts and the dielectric constants. Solvents with high to intermediate dielectric constants better extract polar compounds, and solvents with intermediate to low dielectric constants better extract non-polar compounds (Khoddami et al. 2013). The extraction yield using water and ethanol was the highest (Table 1). The extraction yield using the solvent ethyl acetate was intermediate, and approximately fivefold lower than the aqueous-ethanolic extraction. The yield using petroleum ether was the lowest, and this extract was not used in the experimental treatments due to the very low yield.

**Table 1.** Features of the extraction procedures and data of standardization of extracts to betulinic acid.

Solvent	Extract	Dielectric constant	Yield in dried extract (%)	Betulinic acid (mg/g)	Total betulinicacid extracted (mg)^a^
Water:ethanol (1:8)	AEE	30.78	4.30 ± 0.30	4.60 ± 2.50	98.90 ± 7.00
Ethyl acetate	EAE	6.02	0.70 ± 0.03	107.60 ± 5.00	360.50 ± 12.00
Petroleum ether	PEE	4.03	0.06 ± 0.01	0	0

a Extracted from starting plant material 500 g.

**Figure 2.**
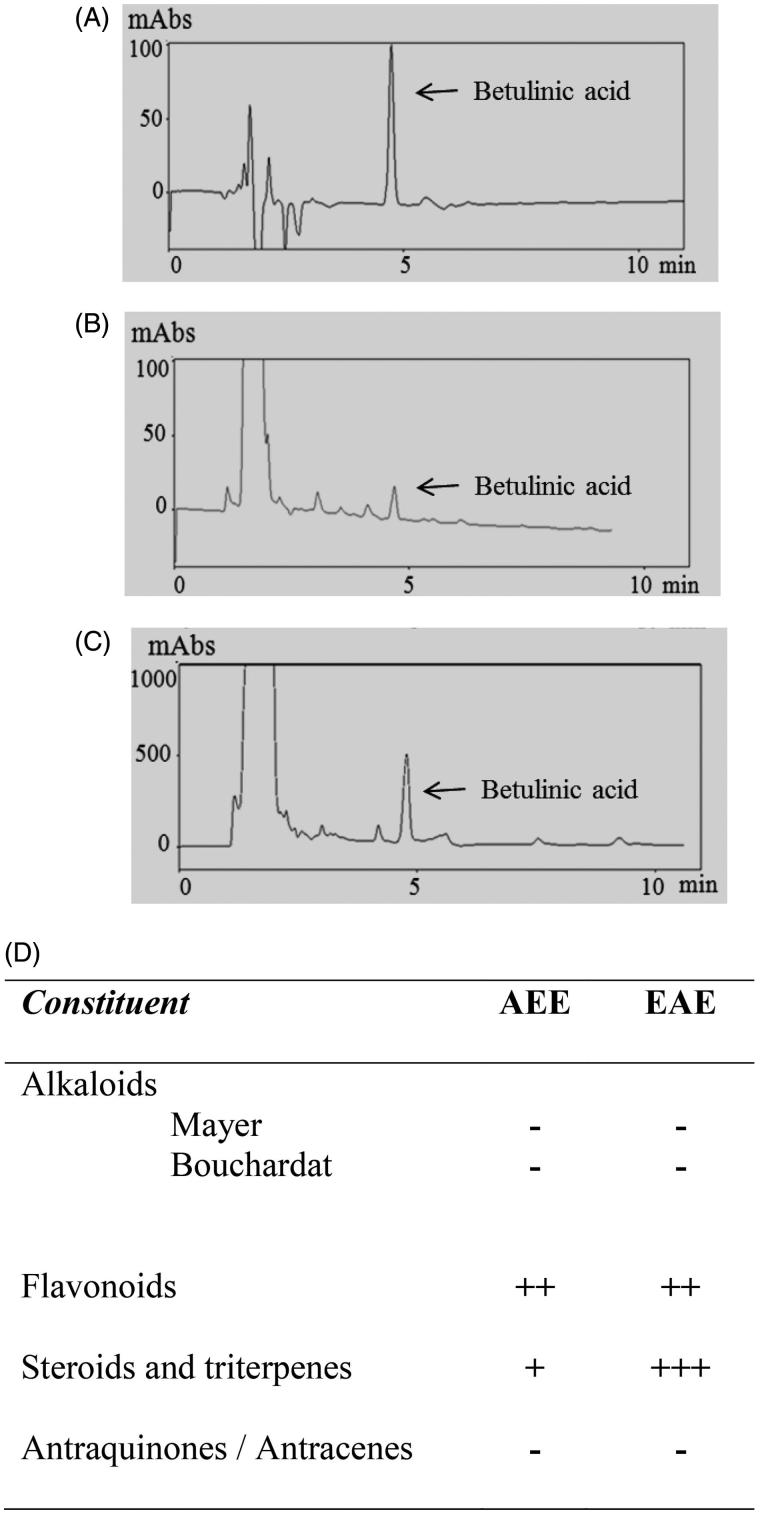
HPLC chromatograms of betulinic acid (0.2 mg/mL) of (A) analytical standard grade, (B) the AEE at 5 mg/mL and (C) the EAE at 10 mg/mL with peaks corresponding to betulinic acid at 4.6 min. (D) Phytochemical screening of AEE and EAE, where (+/++/+++) corresponds to light, moderate and intense positive reactions (presence of the constituent), respectively, and (−) corresponds to a negative reaction (absence of the constituent).

Table 1 and Figure 2(A–C) show the quantification data of betulinic acid. Figure 2(A) shows the chromatogram of betulinic acid of analytical standard grade and the major peak corresponding to betulinic acid, which appeared at a retention time of 4.6 min. Figure 2(B,C) show the chromatograms of AEE and EAE, respectively. Both chromatograms exhibited peaks that corresponded to betulinic acid. Table 1 shows the calculated concentrations of betulinic acid in AEE and EAE, and the total of betulinic acid extracted from the starting plant material (500 g). The concentration of betulinic acid in EAE was >20-fold higher than AEE (Table 1).

Figure 2(D) shows the phytochemical screening data of extracts. The main difference between AEE to EAE was the content of steroids and triterpenes. This result is consistent with the HPLC data and confirmed that the extraction performed using ethyl acetate was more efficient at extracting triterpenoid compounds, such as betulinic acid. Figure 2(D) shows that considerable amounts of flavonoids were also identified in AEE and EAE.

Ethanol and ethyl acetate are good solvents for betulinic acid (Cheng et al. 2011). The presence of water in the extraction of betulinic acid using ethanol possibly reduced the yield because water increases the dielectric constant of the solvent. Betulinic acid was previously identified as a major compound in the ethyl acetate fraction from the methanolic extract of fruits of *D. indica*. Kumar et al. (2010) registered a similar concentration (97 mg/g) compared to the EAE data obtained in the current work (107.6 mg/g). The current work used a one-step procedure, and Kumar et al. (2010) used methanolic maceration followed by liquid–liquid fractionation.

**Figure 3.**
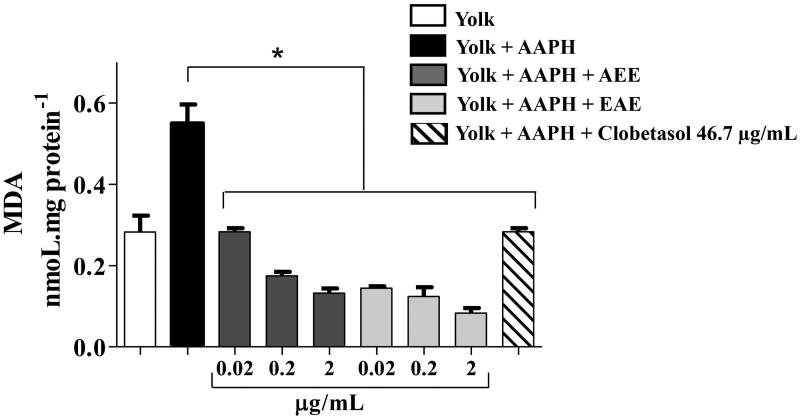
Prevention of lipid peroxidation *in vitro* by the AEE and the EAE from the fruit of *D. indica.* (*) denotes significant difference compared to the control, in which egg yolk lipids were treated with the free radical-generating compound 2,2′-azobis(2-methylpropionamidine) dihydrochloride (AAPH), *p* < 0.001. Data are the means of three independent experiments.

The increased generation of reactive species leads to the molecular damages observed in inflammatory processes, such as in psoriasis (Kadam et al. 2010). Some *in vivo* studies in rats demonstrated that betulinic acid attenuated oxidative stress in experimental inflammation models (Lingaraju et al. 2015). Therefore, the promising wound-healing properties of AEE and/or EAE may be associated with some induced protection against oxidative damage. The extracts were screened initially for protection against *in vitro* lipid peroxidation induced by AAPH from lipids in egg yolk (Figure 3). AEE and EAE provided significant protection against lipid peroxidation and neutralized AAPH at concentrations as low as 0.02 μg/mL (*p* < 0.001). EAE exhibited subtly more protection at 2 μg/mL.

**Figure 4.**
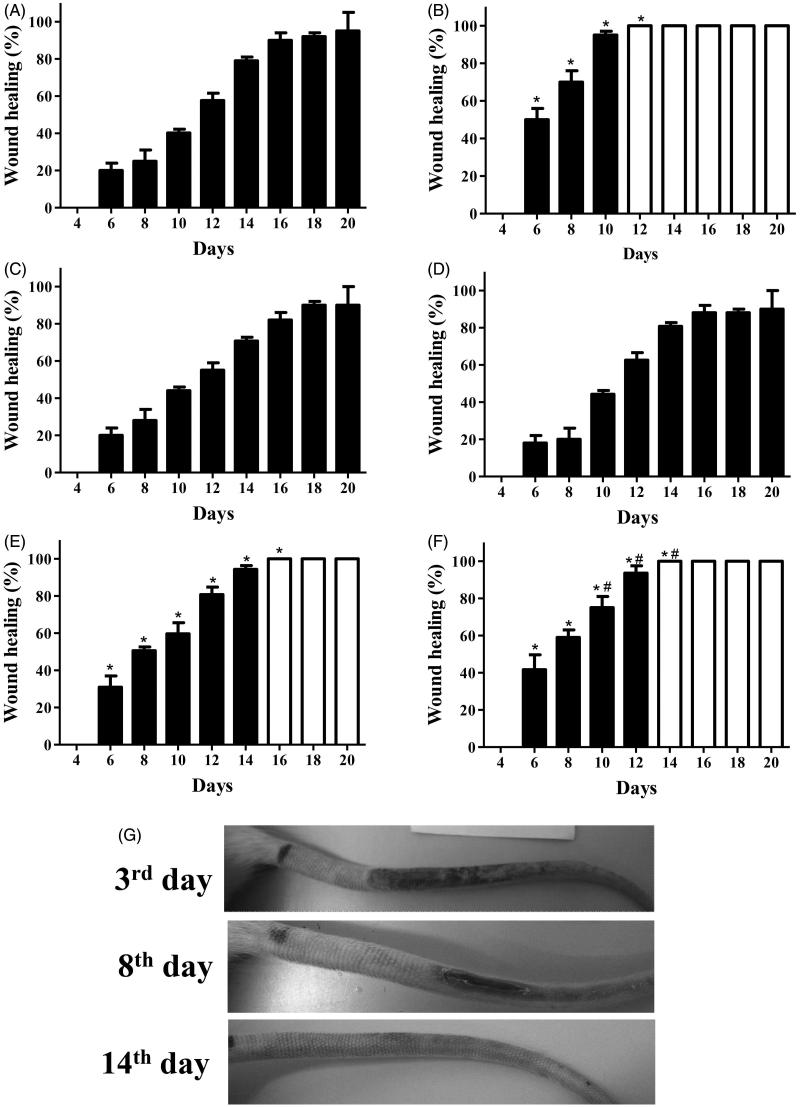
(A–F) The wound-healing effect of AEE and EAE from the fruit of *D. indica* applied topically on radiation-induced psoriasis-like wounds in rats and the control groups. Closed bars correspond to wounds during the repair process, and open bars correspond to completely healed wounds. The negative control was treated only with (A) the excipient (1 mL of ethanol:water 1:2), and the positive control was treated with (B) clobetasol propionate at 0.5 mg/mL. AEE and EAE were applied at (C/D) 5 mg/mL and (E/F) 50 mg/mL, respectively. The evolution of wound healing in the tails of rats treated with EAE at (G) 50 mg/mL; wounds healed on day 14 in this group. (*) denotes significant difference compared to the negative control, *p* < 0.001. (#) denotes significant difference compared to AEE treatment at 50 mg/mL, *p* < 0.05. Data are means of six mice from each group (biological replicates).

**Figure 5.**
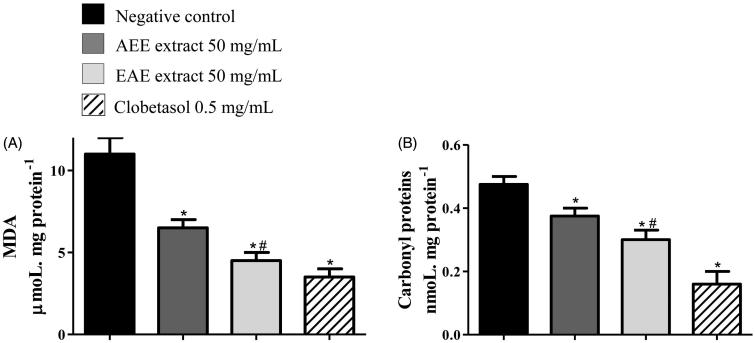
Protection against oxidative damage in the tail skin of rats with radiation-induced wounds that were treated with 50 mg/mL of AEE or EAE from the fruit of *D. indica* and the control groups. The positive control was treated with clobetasol propionate (0.5 mg/mL), and the negative control was treated only with the excipient (1 mL of ethanol:water 1:2). The analysis was performed in technical triplicates with tissue samples of six mice from each group (biological replicates). Protection against (A) lipid peroxidation and (B) protein carbonylation. (*) denotes significant difference compared to the negative control, *p* < 0.001. (#) denotes significant difference compared to AEE treatment, *p* < 0.05.

Figures 1,4–5 show the *in vivo* effects on radiation-induced rat tail wounds. Figure 4(A–F) shows bar diagrams of wound healing over time using macroscopic examinations. Closed bars correspond to wounds during the repair process, and open bars correspond to completely healed wounds. Data of the negative controls (Figure 4(A)) show that wound healing was prolonged in this group. Wound healing in the negative control was nearly 40% on day 10, and not all animals achieved complete healing on day 20. Figure 4(C,D) shows that AEE or EAE treatments at 5 mg/mL did not accelerate wound healing. However, treatments with clobetasol at 0.5 mg/mL (Figure 4(B)), AEE at 50 mg/mL (Figure 4(E)) or EAE at 50 mg/mL (Figure 4(F)) induced significant acceleration of wound healing. EAE was the extract that exhibited the most promising wound-healing activity (Figure 4(F)). The activity of EAE was closer to clobetasol. EAE at 50 mg/mL produced wound healing (nearly 60%) on day 8, and wounded tissues had repaired completely on day 14 (Figure 4(F)). Figure 4(G) illustrates the evolution of wound healing in rats treated with EAE (50 mg/mL). Wound healing in rats treated with AEE at 50 mg/mL was nearly 60% on day 10, and apparent complete repair was registered on day 16 (Figure 4(E)). Only samples of skin tissue from animals treated with 50 mg/mL extracts were subjected to the following evaluations because of the ineffectiveness of AEE and EAE at 5 mg/mL.

Figure 1 shows the microscopic evaluation data (histology). The Perry’s scientific tail model uses UV radiation to induce skin wounds and inflammation. This model cannot reproduce all of the dysfunctions of keratinocytes and the immune-mediated processes observed in psoriasis, but it is accepted as a screening method for assessments of anti-psoriatic activity because it increases parakeratosis and reduces orthokeratosis. An induction of orthokeratosis in areas of scale of epidermis (between the follicles) can be taken as an indicator of anti-psoriatic activity (Hofbauer et al. 1988; Vijayalakshmi et al. 2012). The microscopic findings revealed that EAE at 50 mg/mL restrained parakeratosis compared to orthokeratosis in up to 45% (Figure 1(A)), whereas clobetasol did in nearly 55% compared to the negative control (*p* < 0.001). AEE restrained parakeratosis compared to orthokeratosis in nearly 25% (Figure 1(A)). EAE produced superior reductions in immune cell infiltration in granulation tissue compared to AEE (*p* < 0.05), and these results were closer to the effects of clobetasol (Figure 1(B)). Figure 1(C) shows an image of a slice of tissue from a positive control showing no parakeratosis and, predominant orthokeratosis instead (score 1). Figure 1(F) shows an image of a tissue slice of a negative control with intense parakeratosis compared to orthokeratosis (score 4) and swollen epidermal cones with inflammatory infiltrate.

Figure 5 shows the biomarkers of oxidative damage in biomolecules of tail skin from rats subjected to the experimental treatments. These evaluations were performed to verify whether the wound-healing effect was associated with a potential protection of the extracts against oxidative damage *in vivo*. Two biomarkers, which normally increase during inflammation, were assessed: lipid peroxidation, which was a marker of damage on membrane lipids (Zhao et al. 2014), and the concentration of carbonyl proteins, which was a marker related to protection against protein modifications. Figure 5(A,B) shows that EAE induced significantly superior protection (*p* < 0.05), closer to clobetasol. Tissue from mice treated with EAE exhibited reduced lipid peroxidation (up to 60%) and protein carbonylation (up to 40%) compared to the negative control (*p* < 0.001).

## Conclusions

This study supports the traditional claims of the anti-inflammatory activity of *D. indica*. The results suggested that fruit extracts accelerated the healing of psoriasis-like wounds and reduced inflammation via a mechanism associated with protection against oxidative damage in biomolecules. EAE exhibited the most promising activity, and this extract contained the highest concentration of betulinic acid. These results suggest that betulinic acid may be an active constituent, and further studies should evaluate the activity of betulinic acid for the treatment of psoriasis.
